# An Automated Optimal Engagement and Attention Detection System Using Electrocardiogram

**DOI:** 10.1155/2012/528781

**Published:** 2012-08-09

**Authors:** Ashwin Belle, Rosalyn Hobson Hargraves, Kayvan Najarian

**Affiliations:** Department of Computer Science, School of Engineering, Virginia Commonwealth University, 401 West Main Street, P.O. Box 843019, Richmond, VA 23284-3019, USA

## Abstract

This research proposes to develop a monitoring system which uses Electrocardiograph (ECG) as a fundamental physiological signal, to analyze and predict the presence or lack of cognitive attention in individuals during a task execution. The primary focus of this study is to identify the correlation between fluctuating level of attention and its implications on the cardiac rhythm recorded in the ECG. Furthermore, Electroencephalograph (EEG) signals are also analyzed and classified for use as a benchmark for comparison with ECG analysis. Several advanced signal processing techniques have been implemented and investigated to derive multiple clandestine and informative features from both these physiological signals. Decomposition and feature extraction are done using Stockwell-transform for the ECG signal, while Discrete Wavelet Transform (DWT) is used for EEG. These features are then applied to various machine-learning algorithms to produce classification models that are capable of differentiating between the cases of a person being attentive and a person not being attentive. The presented results show that detection and classification of cognitive attention using ECG are fairly comparable to EEG.

## 1. Introduction

In today's high-paced, hi-tech, and high-stress environment, a common sufferer is our cognitive processing and capacity. Cognitive psychology primarily deals with people's ability to acquire, process, and retain information which is a fundamental necessity for task execution [1]. Quality of task performance largely depends on the individual's capacity to inculcate and sustain high levels of engagement and attention during cognitive activities. However, considering the perils of modern lifestyles such as extended work hours, long to-do lists, and neglected personal health coupled with repetitious nature of daily activities and professions, sleep deprivation and fluctuating attention levels as well are becoming a commonplace issue that needs to be tackled. Momentary or prolonged lapse of attention for certain critical professions such as doctors, pilots, defense personnel, and road transportation drivers can be catastrophic and sometimes deadly.

Studying alertness and drowsiness is not a new domain in scientific research. Numerous research areas are actively studying the concepts of attention, alertness, distraction, and drowsiness. Many of these researches focuses on nonsensory mechanisms to identify and quantify levels of attention in individuals [2–5] such as user's daily routine, schedules, activities, with self-reports from users describing patterns in activities and attention levels and so forth. More recently researchers have begun using biosignals to understand the complex implication of cognitive processing on physiological parameters. Electroencephalogram (EEG) is a popular example of a physiological signal that researchers use extensively in understanding cognitive functioning [6–8]. The use of EEG for detecting and identifying attention/focus in individuals is an established concept. Several concepts have been developed for improving concentration and other cognitive functions of both attention-related disorder and head trauma patients [9–11]. However, there are some fundamental issues regarding the procedure of collecting EEG. It requires the individual to wear a head gear which can be disruptive and troublesome for long-duration usage. The EEG electrode sensors also need to be moistened with electrode gel which can be uncomfortable for the user at the contact points on the scalp. Also, the EEG collection device is usually not designed to be portable; they tend to be slightly large fixed devices which make the collection of EEG confined to a set of environmental contingencies. Furthermore, the EEG signal itself is highly sensitive to noise. Movement of the muscles around the scalp, movement of the subject, talking, blinking, and so forth can induce various unwanted artifacts into the signal thereby disrupting the quality of neuroelectric information contained within the signal.

For this reason, this research is attempting to use Electrocardiogram (ECG) for detecting cognitive attention in individuals. The ECG is a fundamental physiological signal which can be collected easily with a tiny wearable and portable monitor. Since the collection device is portable and has a small footprint on the body, it allows the capture of ECG signals from individuals in various situations in a noninvasive manner. The portability of such a data collection unit allows a more realistic study of human cognitive activities during task execution under various circumstances. The research presented in this paper is attempting to establish a correlation between cognitive attention and its implications on ECG. By being able to identify a pattern and correlation between the two it becomes possible to predict well in advance, an individual's potential loss of attention and ingression of sleepiness during a task execution. This also provides the ability for preemptive feedback to the user upon identifying diminishing attention levels and thereby improving the individuals' overall performance.

The rest of this paper is organized as follows: Section 2 describes the experimental setup, followed by a description of methods in Section 3.  Section 4 describes the results and conclusion of this research.

## 2. Experimental Setup

An essential aspect of this research has been the collection of the data itself. Extensive search revealed that there was no dataset available, freely or otherwise, which catered to the exact needs to this particular study. Since the study is about utilizing ECG collected via a portable armband to detect the presence or lack of attention/focus in an individual, the dataset had to be collected specifically based on the requirements of this research.

In the designed experiment, volunteer subjects were individually asked to watch a series of preselected video clips during which two physiological signals, that is, the ECG and EEG, were acquired. Based on their content, the chosen video clips fell in either of two categories that is either “interesting” or “noninteresting,” requiring high and low levels of viewer engagement, respectively. The average length of each selected video clips was about 4-minute long. For each category the respective video clips were put together to form a video montage of about 20-minute viewing duration. The first category of the video montage named “interesting” included engaging scenes from documentaries, popular movie scenes, high-speed car chases, and so forth. which were intended to keep the viewers attentive and engaged with its content. The second video montage named “noninteresting” contained videos which were repetitive and monotonous in nature such as a clock ticking and still images shown for extended periods of time. These were intended to induce boredom in subjects and thereby reduce their attentiveness. Viewing the two categories of video montages one after the other required contrasting levels of engagement and focus from the participant, thereby ensuring (as far as possible) that the subjects were interested and paid attention to the interesting video set and the subjects were subsequently bored and lost focused attention during the noninteresting videos.

During the experiment the ECG signal was collected using the SenseWear-Pro armband developed by Bodymedia Inc. This armband is capable of collecting ECG data at 128 Hz [12].

**Figure 1:**
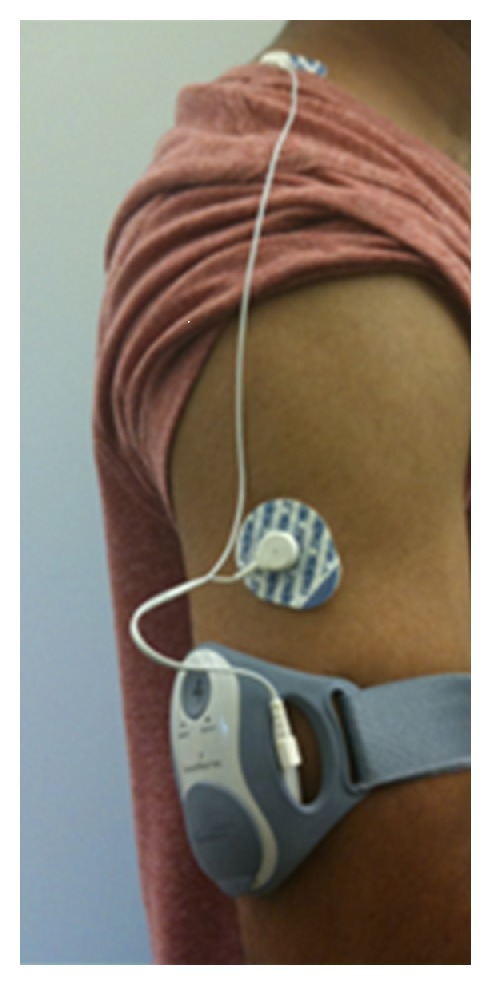
Two leads ECG collection from Armband.

As shown in Figure 1, two leads from the armband are attached to the subject using ECG adhesive electrodes patches. One lead of the leads is placed on the side of the arm and the other lead is fastened on the bridge between the neck and shoulder.

The EEG signal was collected from the subjects using MP150: EEG-100C a product by Biopac Inc. With this system an EEG cap is provided that fits snug on the head of the subject and it collects the EEG signal at a sampling rate of 1000 Hz. Signals were collected from the forehead or the frontal cortex (fp1 and fp2) with a ground reference from the ear lobe. The frontal cortex is primarily responsible for attention and higher-order functions including working memory, language, planning, judgment, and decision-making [13]. The entire setup is completely noninvasive and only utilizes surface contact sensors. The data collection has been conducted with required IRB approval.

## 3. Methods

**Figure 2:**
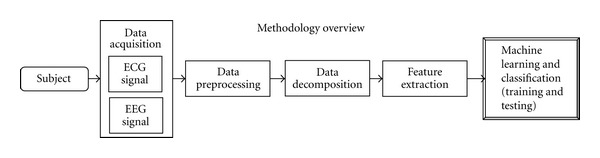
Methodology overview.

The schematic diagram in Figure 2 illustrates the overall method of this study. As shown the two physiological signals ECG and EEG are acquired from the subject during the experiment.

The acquired raw signals are first preprocessed to remove unwanted artifacts presented within the signals. Next the preprocessed signals are decomposed using various decomposition and analysis methods. In the next step valuable and informative features are extracted from the decomposed components of the signal. These extracted features are finally fed to the machine-learning step where classification models are developed to classify the feature instances to either of two cases “attention” or “nonattention.”

### 3.1. Data Preprocessing

The acquired raw ECG signal contains some inherent unwanted artifacts that need to be dealt with before any analysis can be performed on it. The cause of these artifacts, which is usually frequency noise or baseline trend, could be due to a number of reasons such as subjects' movement causing motion artifacts, breathing patter artifact, loose skin contact of the electrodes, and electric interference (usually found around 55 Hz). Therefore a preprocessing step has been designed to ensure that the signal is as clean and artifact free before analysis.

#### 3.1.1. ECG Preprocessing

**Figure 3:**
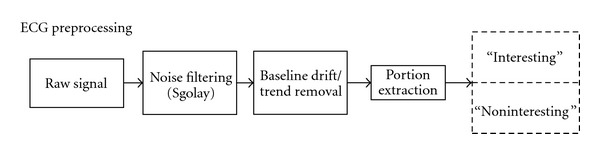
ECG preprocessing.

The preprocessing steps for the ECG signal are shown in Figure 3. Since each signal has to be filtered differently based on the type of inherent noise, the raw ECG signal is first filtered using “SGolay” filtering method. The “SGolay” filter was developed by Savitzky-Golay. This filter is a digital polynomial filter based on least square smoothing mechanism. The SGolay filters are typically used to smooth out a noisy signal with a large frequency span. They perform better than standard averaging FIR filters, since these filters tend to retain a significant portion of the signals high-frequency content while removing only the noise [14].

Next, the filtered ECG data is sent through a baseline drift removal step. Typically baseline drift is observed in ECG recordings due to respiration, muscle contraction, and electrode impedance changes due to subject's movement [15]. To remove the baseline drift first the regression line that best fits the samples within a window of size equal to the sampling rate is determined.

Given *n* points of the ECG signal (*x*_1_, *y*_1_), (*x*_2_, *y*_2_),…, (*x*_*n*_, *y*_*n*_), the best fit line associated with these points can be computed as follows:

(1) m=n(∑1nxy)−(∑1nx)(∑1ny)n(∑1nx2)−(∑1nx)2,b=∑1ny−m(∑1nx)n,y=mx+b,

where *y *is a point on the line, *m* is the slope of the line, and *b* is the intercept. The computed best fit line for each window is then subtracted from the original signal window to obtain a baseline drift-free signal.

After the raw ECG signal has been filtered of noise and baseline drift, the signal is then split into two portions based on the acquisition and experiment framework. The two portions of signals, namely, “interesting” and “noninteresting” are extracted from the original signal using timestamps that are recorded and indexed during signal acquisition. Splitting and analyzing the two sections of data separately facilitate supervised learning mechanism during the training phase in the machine learning step.

#### 3.1.2. EEG Preprocessing

**Figure 4:**
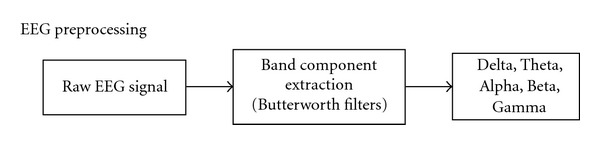
EEG preprocessing steps.

The EEG signal is comprised of a complex and nonlinear combination of several distinct waveforms which are also called band components. Each of the band components is categorized by the frequency range that they exist in. The state of consciousness of the individuals may make one frequency range more pronounced than others [16]. As shown in Figure 4, the different band components are extracted from the raw EEG signal using Butterworth bandpass filters. Five primary bands of the EEG signal are extracted, namely, Delta (0.2–4 Hz), Theta (4–8 Hz), Alpha (8–13 Hz), Beta (13–30 Hz), and Gamma (30–55 Hz).

### 3.2. ECG Decomposition: Using Stockwell Transform

The *S*-transform was proposed by Stockwell and his coworkers in 1996. The distinction of *S*-transform is that it produces decomposition of frequency-dependant resolution in the time-frequency domain while entirely retaining the local phase information. In other words, the *S*-transform not only estimates the local power spectrum, but also the local phase spectrum, which is highly desirable in studying complex physiological signals such as the ECG.

When it comes to analyzing dynamic spectrum or local spectral nature of nonstationary observations such as the ECG some of the popular methods include Short-Time Fourier Transform (STFT) [17], Gabor transform [18], complex demodulation [19] which produces a series of band pass filtered voices and is also related to the filter bank theory of wavelets and so forth. Some methods represent the transformation in a combination of time and frequency domain such as the Cohen class [20] of generalized time-frequency distributions (GTFD), Cone-Kernel distribution [21], Choi-Williams distribution [22] as well as the smoothed pseudo Wigner distribution (PWD) [23]. One of the more popular methods for decomposition and analysis in time-frequency domain is Wavelet Transform. Discrete Wavelet Transform or DWT performs decomposition of a signal that provides excellent time resolution while maintaining key spectral information or frequency resolution [24, 25].

Although *S*-transform is similar to wavelet transform in having progressive resolution, unlike wavelet transform, the *S*-transform retains absolutely referenced phase information. Absolutely referenced phase implies that the phase information calculated by the *S*-transform is referenced to time *t* = 0, which is also true for the phase given by the Fourier transform. The only difference being the *S*-transform provides the absolute referenced phase information for each sample of the time-frequency space.

#### 3.2.1. Mathematical Formulation of *S*-Transform

There are two varieties of *S*-transform, continuous and discrete. The continuous *S*-transform [26] is essentially an extension of the STFT. It can also be seen as a phase-corrected format of the Continuous Wavelet Transform (CWT).

The STFT of a signal *h(t)* is defined as

(2) STFT(τ,f)=∫−∞∞h(t)  g(τ−t)e−j2πftdt,

where

- *τ*  is the time of spectral localization,
- *f* is the Fourier frequency,
- *g*(*t*) denotes a window function.

The *S*-transform can be derived from the above STFT equation simply by substituting the window function *g*(*t*) the Gaussian function:

(3) g(t)=|f|2πe−(t2f2)/2.

Therefore the *S*-transform be mathematically defined as follows:

(4) $$\begin{array}{c} S\left(\tau ,f\right)=\int_{-\infty }^{\infty }h\left(t\right)\;\frac{\left|f\right|}{\sqrt{2\pi }}e^{-\left({\left(\tau -t\right)}^{2}f^{2}\right)/2}e^{-j2\pi ft}dt. \end{array}$$

Since *S*-transform essentially functions with the Gaussian window during decomposition, it can be deduced that with a wider window in the time domain the transformation can provide better resolution for lower frequency, and with a narrow Gaussian window the resolution for higher frequency is better accentuated.

For application of *S*-transform in this study, the continuous *S*-transform does not prove to be a practical choice. Simply because the acquisitions of the ECG signal itself were performed with discrete sampling and also a continuous decomposition of this signal for all frequencies can be extremely time consuming, thereby not computationally pragmatic. Hence a Discrete version of the *S*-transform has been adopted for the decomposition of the ECG signal.

The discrete *S*-transform can be presented as follows.

Let *h*[*kT*] be the discrete time series signal to be investigated, where *k* = 0, 1,…, *N* − 1, and *T* is the time sampling interval. The discrete format of the Fourier transform can be shown as follows:

(5) H[nNT]=1N∑k=0N−1h[kT]e2jπnk/N.

Using the continuous *S*-transform equation and the above equation, the time series, *h*[*kT*]'s *S*-transform can be represented as follows: (making *f* → *n*/*NT*and *τ* → *jT*)

(6) S[jT,nNT]=∑m=0N−1H[m+nNT]e2π2m2/n2e2jπmj/N, n≠0,

where *j*, *m*, and *n* = 0, 1,…, *N* − 1.

#### 3.2.2. Application of *S*-Transform

**Figure 5:**
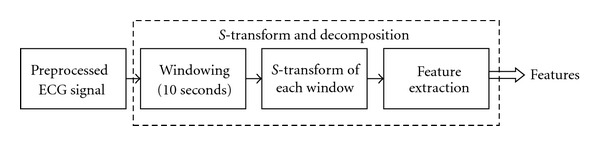
*S*-Transform application on ECG signal.

Figure 5 shows the different steps involved in the decomposition of the ECG signal using *S*-transform. First, the preprocessed ECG signal is sent through a windowing mechanism. In this mechanism, the preprocessed ECG signal is partitioned into tiny windows. These windows are nonoverlapping and contain ECG data of 10 sec interval (128 Hz ∗ 10 sec = 1280 data-points/window).

After the windowing step, each of the 10 seconds windows is decomposed using *S*-transform. The output of the *S*-transform is a complex 2-dimensional matrix with rows representing the frequencies and the columns represent the time values. The *S*-transform algorithm applied in this study is tuned to produce a stepwise frequency range with step size being 1 Hz and the time interval between samples in the result is 1 step unit.

**Figure 6:**
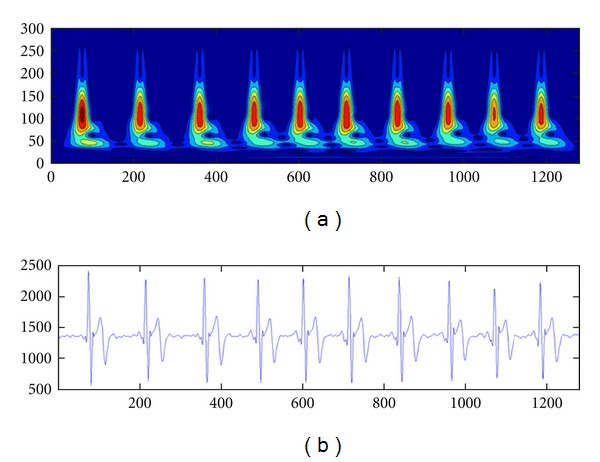
(a) shows the contour-based visualization of frequency spectrum along time, based on the *S*-transform of the signal window. (b) shows the original signal window.

An example output of a 5-second window of an ECG data after *S*-transform is given in Figure 6.

Figure 6 shows the exact point-to-point representation of the original (Figure 6(b)) signal in the *S*-transforms time-frequency domain. The *S*-transform output matrix has been shown in a contour map display (Figure 6(a)).

#### 3.2.3. Feature Extraction

The output of each window is a frequency-time represented matrix. Each instance of the matrix is frequency point and a time point (by the row and column position, resp.). So the entire output matrix can be presented as follows: ST(*x*, *y*), where *x* is the frequency (row) location and *y* is the time (column) location.

The extraction of features from the derived output matrix of ST is performed in two steps. In the first step the output matrix is reduced from two dimensions to a single dimension. This is done by computing certain statistical measures along the frequency dimension *x*, while retaining the discreteness in the time dimension *y* as is. The computed statistical measures along frequencies (*f*) are as follows:

- mean of frequencies (*f*),
- sum of frequencies (*f*),
- product of frequencies (*f*),
- standard Deviation of frequencies (*f*),
- range (*f*).

At the end of the first step we get an array of features from the frequency domain as follows:

(7) Freqfets=[mean(f),sum(f),product(f),std(f),    range(f)].

The next step is to compute statistical features along the time domain.

(i) Mean:
  (8) mean(ST)=mean(fi), where  fi∈Freqfets.
(ii) Sum:
  (9) sum(ST)=sum(fi), where  fi∈Freqfets.
(iii) Mean of autocovariance:
  (10) mean(autocovariance(ST))=mean(autocovariance(fi)),
  where  *f*_*i*_ ∈ Freq_fets_.
(iv) Sum of cross-correlation:
  (11) sum(autocorrilation(ST))=sum(autocorrelation(fi)),
  where  *f*_*i*_ ∈ Freq_fets_.
(v) Log_2_ of Variance:
  (12) Log2(variance(ST))=Log2(variance(fi)),
  where  *f*_*i*_ ∈ Freq_fets_.

Two additional features are calculated from the initially obtained ST matrix.

(i) Mean of max frequencies:
  (13) mean⁡(max⁡(ST))=mean⁡(max⁡(ST1,y,ST2,y,…,STx,y)).
(ii) Mean absolute deviation of frequencies:
  (14) $$\begin{array}{c} \text{mean}\left(\text{abs}\left(\text{ST}\right)\right)=\text{mean}\left(\text{abs}\left(\text{ST}-\text{mean}\left(\text{ST}\right)\right)\right). \end{array}$$
  After the feature extraction has been performed, the total feature set for the *S*-Transform step will contain (5 (features in step 1) ∗ 5 (features in step 2)) + 2 (additional noniterative features) = 27 (features columns per window).

### 3.3. EEG Decomposition and Analysis: Using Wavelet Transform

The EEG signal exhibits complex behavior and nonlinear dynamics. In the past wide range of work has been done in understanding the complexities associated with the brain through multiple windows of mathematics, physics, engineering and chemistry, physiology, and so forth [27, 28]. The intention of acquiring and analyzing EEG in this research is to develop a benchmark of sorts for attention recognition. The key point of this study is to see if the ECG signal that can be collected from a portable armband can be comparably efficient in recognizing an individual's attention and focus.

The small yet complex varying frequency structure found in scalp-recorded EEG waveforms contains detailed neuroelectric information about the millisecond time frame of underlying processing systems, and many studies indicate that waveform structure at distinct scales holds significant basic and clinical information [29, 30]. Small-scale neural rhythms, in particular event-related oscillation EROs, have been regarded as fundamental to perception and cognition [29]. Wavelet analysis provides a powerful method of isolating such rhythms for study. There are several applications of wavelet transform on EEG analysis. It has been used in removal of noise from raw EEG waveforms since wavelet coefficients facilitate the precise noise filtering mechanism by zeroing out or attenuating any coefficients associated primarily with noise before reconstructing the signal with wavelet synthesis [31–33]. Wavelet analysis of EEG has also been extensively used for signal processing applications in intelligent detection systems for use in clinical settings [34, 35]. Wavelet transform has also been used for compression EEG signals. Wavelet compression techniques have been shown to improve neuroelectric data compression ratios with little loss of signal information [36, 37]. It can also be seen for component and event detection as well as spike and transient detection within the EEG waveforms. Wavelet analysis has proven quite effective in many research studies [33–38].

#### 3.3.1. Mathematical Formulation of Wavelet Transform

Wavelet transforms essentially exist in two distinct types: the Continuous Wavelet Transform (CWT) and the Discrete Wavelet Transform (DWT). In this study for the analysis of the EEG signal the DWT method has been employed. The advantages of using DWT is that it allows the analysis of signals by applying only discrete values of shift and scaling to form the discrete wavelets. Also, if the original signal is sampled with a suitable set of scaling and shifting values, the entire continuous signal can be reconstructed from the DWT (using Inverse-DWT). A natural way of setting up the parameters *a* (scaling) and *b* (shifting) is to use a logarithmic discretization of the *“a”* scale and link this, respectively, to the step size taken between “*b”* locations or shifts. To link “*b”* to “*a”* discrete steps are taken to each location “*b,”* which are proportional to the “*a”* scale. This kind of mother wavelet can be shown in the following form.

Discrete mother wavelet representation:

(15) Ψm,n(t)=1a0m(t−nb0a0ma0m),

where

- integer's *m* and *n* control the wavelet shifting and scaling, respectively,
- *a*_0_ is a specified fixed dilation step parameter set at a value greater than 1,
- *b*_0_ is the location parameter which must be greater than zero.

Analysis equation (DWT):

(16) Wmn=∫−∞+∞x(t)Ψmn∗(t)dt.

Synthesis equation (inverse DWT):

(17) x(t)=c∑m∑nWmnΨmn(t),

where *c* is a constant associated with the mother wavelet.

#### 3.3.2. Application of DWT on EEG

In this study, Discrete Wavelet Transform or DWT is applied to the EEG band components which are extracted in the preprocessing step.

**Figure 7:**
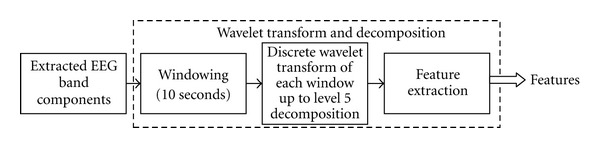
EEG decomposition and analysis steps using wavelet transform.

As shown in Figure 7, each of the extracted band components is sent through the “windowing” step. In this step the interesting and boring portions of the band components based on the timestamps of the original EEG are extracted and sent through a windowing mechanism. In this mechanism, each band component signal is partitioned into tiny windows. The windows are 10-second long and are nonoverlapping. The EEG signal is acquired at a sampling rate of 1000 Hz, so each window will have 1000 Hz ∗ 10 sec = 10000 data points.

Each window is then decomposed using DWT. Performance of the Wavelet transform depends on the mother wavelet chosen for decomposition of the signal. A common heuristic is to choose one similar to the shape of the signal of interest. So for the set of band components that is extracted from the original EEG signal different mother wavelets that suit different bands are applied during decomposition.

**Figure 8:**
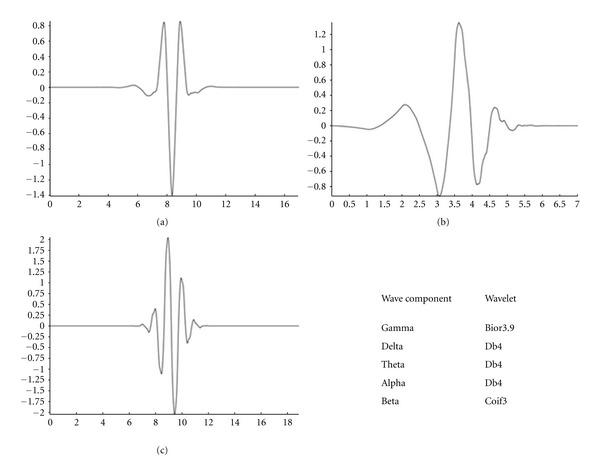
(a) “COIF3” wavelet, (b) “DB4” wavelet, and (c) “BOIR3.9” wavelet.

As shown in Figure 8, the analysis of the Gamma wave component, the mother wavelet chosen is the “bior3.9” from the bi-orthogonal family of wavelets. Delta, Theta, and Alpha wave components are decomposed using “db4” as their mother wavelet from the Daubechies family of wavelets. Finally Beta waves are decomposed using “coif3” as the mother wavelet from the Coiflets wavelet family. These wavelets were chosen not only based on the shape and complexity but also because they seemed to be commonly used for such application in related research.

The decomposition process in wavelet transform can be performed iteratively into several levels. The number of levels chosen for decomposition is application specific and also depends on the complexity of the signal. For window of the EEG signal band components, 5 levels of decomposition seemed to provide all the required useful information; further decomposition did not yield a better result. The detailed coefficients of all the stages from 1 through 5 and the approximation coefficient of level 5 are retained for feature extraction step.

#### 3.3.3. Feature Extraction Step

The features computed from these coefficients are as follows. (Here, (*x*_1_, *x*_2_,…, *x*_*n*_) represents the values of each coefficient from the 10 sec window.)

(i) Standard deviation:
  (18) std=    1n  ∑i=1nxi2.
(ii) Entropy: entropy is a statistical measure of randomness. It is very useful in evaluating the information present within a signal:
  (19) entropy=−sum(p∗log⁡2(p)),
  where *p* is the histogram of the signal.
(iii) Log of variance: let the probability mass function of each element be as follows *x*_1_ ↦ *p*_1_,…, *x*_*n*_ ↦ *p*_*n*_, then
  (20) Variance=∑i=1npi∗(xi−μ)2,
  where *μ* is the expected value, that is,
  (21) μ=∑i=1npi∗xi.Therefore,Log  of  variance=log⁡2⁡(Variance(x)).
(iv) Mean of frequencies (discrete Fourier domain):
  (22) dft(xk)=∑k=1N−1X(j)ej(2π/N)kn,
  where a net of *N* time samples, dft(*x*_*k*_), represents the magnitude of sine and cosine components in the samples given by *e* ^*j*(2*π*/*N*)*kn*^:
  (23) mean  of  fourier  domain=mean(dft(x)).
(v) Variance of probability distribution:
  (24) Probability  Distribution  Function=  P[a≤x≤b]=∫abf(x)dxVariance  of  distribution=variance(P).
(vi) Sum of autocorrelation:
  (25) Autocorrelation  function=R(s,t)=E[(xt−  μ)∗(xt+r−  μ)]σtσs,
  where *s* and *t* are different times in the time series, *μ* is the mean of *X*, *σ* is the standard deviation of *X*, and “*E*” is the expected value operator:
  (26) Sum  of  AutoCorrelation=sum(R(s,t)).
(vii) Mean of autocovariance:
  (27) C(s,t)=E[(xt−  μt)∗(xs−μs)],
  where *s* and *t* are different times in the time series, *μ* is the mean of *X*, and “*E*” is the expected value operator:
  (28) mean  of  autocorrelation=mean(C(s,t)).
  After the feature extraction has been performed, the total feature set for the wavelet transform step will contain; 6 coefficients (5 detailed + 1 approximation) ∗ 7 (features per coefficient) = 42 (features columns per band component). In total there are 5 extracted band components, so, 42 (features per band component) ∗ 5 (different band components) = 210 (total features from EEG). These computed features are then sent to the machine learning stage for classification, training, and testing.

### 3.4. Machine Learning and Classification Model

In this application the result after signal processing on various acquired psychological signals is a large set of features. Since the data was collected in a systematic and controlled environment, the features extracted from respective portions of the signals can be classified under the two presumed categories: “attention” and “nonattention.” Hence supervised learning method is used for this study to developed classification heuristics.

Three different machine learning algorithms have been implemented and tested for this experiment. These are as follows.

#### 3.4.1. Classification via Regression

There are different models for predicting continuous variables or categorical variables from a set of continuous predictors and/or categorical factor effects such as General Linear Models (GLMs) and General Regression Models (GRMs). Regression-type problems are those where attempt is made to predict the values of a continuous variable from one or more continuous and/or categorical predictor variables [28, 38, 39]. This is a nonparametric approach meaning that no distribution assumptions are made about the data whereas in GLM it is either known or assumed that the data follows a specific linear model such as binomial or Poisson. In regression-based classifiers, splits for the decision trees are made based on the variables that best differentiate between the categories of the target classification label variables. Here the decision splits are composed based on regression trees. In regression trees each node is split into two child nodes. As the regression tree grows certain stopping rules are applied to stop the tree growth.

In more general terms, the purpose of the analyses via tree-building algorithms is to determine a set of if-then logical (split) conditions that permit accurate prediction or classification of cases. Tree classification techniques, when applied correctly, produce accurate predictions or predicted classifications based on few logical if-then conditions. Their advantage of regression tree-based classifier over many of the alternative techniques is that they produce simplicity in the output classifier results. This simplicity not only is useful for purposes of rapid classification of new observations but can also often yield a much simpler “model” for explaining why observations are classified or predicted in a particular manner. The process of computing classification and regression trees can be characterized as involving four basic steps: specifying the criteria for predictive accuracy, selecting splits, determining when to stop splitting, and selecting the “right-sized” tree.

#### 3.4.2. C4.5 Classification Method

C4.5 is also a decision-tree-based classification algorithm, developed by Quinlan [39, 40]. It has been developed based on the fundamentals of the ID3 machine-learning algorithm [41]. The C4.5 computes the input data to form a decision tree based on a divide-and-conquer strategy. In C4.5 each node in the tree is associated with a set of cases. Every case is assigned weights to deal with unknown attribute values. At first the entire training set is started off as a root where the weights assigned to all cases are 1.0. From here the tree computes the information gain presented by each attribute of the training set. For discrete attributes the information gain is relative to the splitting of case at every node with distinct values. The attribute with the highest information gain is selected as a test node. After this the divide-and-conquer approach consists of recursively splitting the attributes at each node to form children node based on the information gain of the attribute at each node. C4.5 has been used for several applications in healthcare informatics [42, 43].

#### 3.4.3. Random Forest

Breiman developed random forest classification method which is basically an ensemble classifier that consists of multiple decision trees [44]. It is a very accurate classifier which displays great success with multiple datasets. It is especially useful with data mining extremely large datasets and databases. Unlike the other two mentioned tree-based classifiers random forest uses multiple trees or a forest to develop decisions and classifications. Although in this study it is being used to develop models based on supervised data, random forest can be used for unsupervised data learning as well [45, 46]. Random forest is also popular for applications in biosignal and biomedicine [46].

All of the above-mentioned machine-learning methods are known to have comparable performance to methods such as neural networks in physiological and medical applications [47]. Moreover, methodologies such as neural networks, when analyzed using statistical learning theory, are shown to be susceptible to the issue of overfitting [48–50], hence further encouraging the use of the methods described above, in particular when the number of data or subjects used for training and testing is limited.

In the machine learning step, the three mentioned classifiers are independently implemented on the extracted features of ECG and EEG and the results of each of these classifiers are compared. This is based on a setup developed earlier during initial stages of this experiment. For this experiment ECG signal from 21 subjects and EEG signal from 12 subjects have been collected.

## 4. Results and Conclusion

The classification model for each of the classifiers is developed using “by-subject” or “leave one subject out” based training and test sets. In this type of training and testing, out of the given number of subject say *x*, *x* − 1 subjects are subjects used for training and developing the classification model, while the *x*th subject's data is used for testing the developed model. This procedure is repeated in a round robin fashion until each of the subject's data in the total collected data has been tested with a classification model developed exclusively for it. In this section for each type of classification method used, the average accuracies and other statistics have been presented over all the subjects.

### 4.1. Classification Results of ECG Using *S*-Transform

The results obtained from the analysis and classification of the computed features from Stockwell transform (ST) from the ECG signal are presented.

Table 1 presents the overall average accuracies, specificities, and sensitivities of the three classification algorithms for ECG testing and training models across all subjects.

It can be seen that overall accuracy of random-forest-based classification model was more successful than both C4.5 and classification via regression models with a classification accuracy of nearly 77%.

### 4.2. Classification Results of EEG Using Discrete Wavelet Transform

The features computed from the analysis of the EEG signal using discrete wavelet transform is used to develop different classification models based on the three described classification methods. The results of these classification are presented in Table 2.

Table 2 presents the overall average accuracies, specificities, and sensitivities of the three classification algorithms for EEG testing and training models across all subjects. It can be seen that overall accuracy of random-forest-based classification model was more successful than both C4.5 and classification via regression models with a classification accuracy of nearly 86% for the EEG feature set.

### 4.3. ECG versus EEG Classification Comparison

The results from the ECG feature classification of all three classifier are compared against the classification results of the EEG.

**Figure 9:**
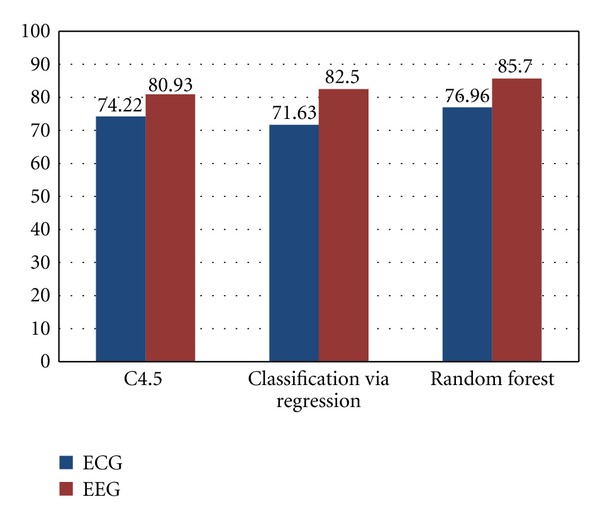
ECG versus EEG classification comparison.

From Figure 9 it can be seen that although EEG inherently has more information to indicate the presence of attention or the lack of it, ECG signal analysis and classification are not very far behind. Random Forest seems to work best for both modalities given an average accuracy of 77% for ECG and 86% for EEG.

## 5. Conclusion

The analysis of the EEG signals is primarily to set a benchmark against which the analysis of the physiological features from the armband can be compared. This system as it has been proposed primarily focuses on the electrocardiogram (ECG) signal and various methods of decomposition are performed on it. The following are the conclusive statements that can be deduced from the systems performance so far.

- It can be seen that to a reasonable level of accuracy the system is able to identify cognitive attention in comparison with that detected by the EEG collected in the same experiment. The focus of this proposal was entirely on ECG alone, and with just this signal it was demonstrated that its classification accuracy was comparable to that of EEG.
- Amongst the various machine learning methods investigated, “classification via regression” seems to perform the best on the combined feature set. However, it was also demonstrated that “random-forest-” based classification works on the subset of features for each different decomposition and analysis method.
- This study also establishes that ECG alone can be used in analyzing cognitive attention and that the fluctuation of attention does have a translated impact on the Cardiac rhythm of an individual.

Here are some of the future work planned to improve the system's classification and prediction performance.

- A larger data set is needed to further validate this experiment. A larger data set is expected to provide a more robust classifier model.
- More novel features are going to be developed and tried for the feature extraction step after decomposition. Having a more diverse base of features usually provides insight into some connate characteristics of the signal which might not be openly evident.
- Feature pruning and other classification methods need to be tried for increasing the accuracy.

## Figures and Tables

**Table 1:** *S*-transform feature classification results of ECG.

*S*-transform feature classification result ECG	Accuracy (average)	Specificity (average)	Sensitivity (average)
C4.5	74.22%	67.31%	81.13%
Classification via regression	71.63%	63.11%	80.15%
Random forest	**76.96%**	66.73%	87.20%

**Table 2:** DWT features classification results of EEG.

DWT feature classification result EEG	Accuracy (average)	Specificity (average)	Sensitivity (average)
C4.5	80.93%	81.11%	80.96%
Classification via regression	82.5%	76.74%	88.26%
Random forest	**85.70** **%**	79.74%	91.66%
